# Conjugation as an evolutionary bottleneck in antimicrobial resistance spread

**DOI:** 10.3389/fmicb.2026.1863866

**Published:** 2026-06-04

**Authors:** Lamarana Jallow, Abdoulie Bojang, Ousman Bajinka

**Affiliations:** 1 Department of Biology, Division of Physical and Natural Sciences, School of Arts and Science, University of The Gambia, Banjul, Gambia; 2 Department of Biochemistry, Cell and Molecular Biology, College of Basic and Applied Sciences, University of Ghana, Legon, Accra, Ghana; 3 West African Centre for Cell Biology of Infectious Pathogens, University of Ghana, Accra, Ghana; 4 MRC Unit The Gambia at the London School of Hygiene and Tropical Medicine, Fajara, Gambia; 5 School of Medicine & Allied Health Sciences, University of The Gambia, Banjul, Gambia

**Keywords:** antimicrobial resistance, conjugation, evolutionary bottleneck, horizontal gene transfer, one health, plasmids, type IV secretion system

## Abstract

Antimicrobial resistance (AMR) is commonly framed as a consequence of mutation and selection, yet this perspective does not fully explain the speed and scale of global resistance dissemination. Here, we argue that AMR is better understood as an amplification problem, in which horizontal gene transfer particularly conjugation governs the spread of resistance genes across bacterial populations and ecological compartments. Conjugative plasmids couple high transfer efficiency with broad host range, enabling rapid dissemination of resistance determinants, including those conferring resistance to last-resort antibiotics. This review synthesizes evidence showing that conjugation is shaped by tightly constrained trade-offs between transfer efficiency, fitness cost, plasmid copy number, and ecological context. These constraints render conjugation a rate-limiting step in dissemination dynamics, such that even modest reductions in transfer efficiency can substantially reduce plasmid persistence and spread. At the same time, plasmids exhibit adaptive features, including compensatory evolution and dynamic regulation of replication, that stabilize their persistence and complicate intervention. This duality positions conjugation as both a central driver of AMR and a tractable therapeutic target. We review emerging strategies to disrupt conjugation, including small-molecule inhibitors, CRISPR-based systems, phage approaches, and ecological interventions, and highlight key challenges related to delivery, evolutionary escape, and real-world implementation. We propose that targeting gene flow rather than gene emergence alone offers a complementary strategy for controlling AMR. By reframing conjugation as a controllable bottleneck in resistance amplification, future interventions may shift the trajectory of AMR from expansion toward containment.

## 1 Introduction

The global acceleration of antimicrobial resistance (AMR) represents one of the most formidable challenges to modern medicine. The recent comprehensive analysis by Murray et al. (2022) estimated that bacterial AMR was directly responsible for 1.27 million deaths in 2019, a toll that exceeds that of HIV/AIDS or malaria (Antimicrobial Resistance Collaborators, 2022). This crisis, however, cannot be fully explained by the canonical paradigms of mutation and clonal expansion alone (Arredondo-Alonso et al., 2025; Koh et al., 2025). Despite decades of antibiotic development and stringent stewardship efforts, resistance to last-resort antibiotics has emerged and disseminated with a speed that outstrips the capacity of *de novo* mutation to generate it (O’Neill, 2016). The starkest examples are the near-instantaneous globalization of key resistance genes. Carbapenemases of the NDM family, first identified in a single patient in India in 2008, were detected on six continents within five years. Similarly, the mobilized colistin resistance gene *mcr-1* emerged in China in 2015 and was globally distributed across human, animal, and environmental sectors by 2017 (Liu et al., 2016; Wang et al., 2018). This epidemiological velocity points to a process fundamentally different from the stepwise accumulation of chromosomal mutations under localized selection.

We propose that antimicrobial resistance is best understood as an amplification crisis, a problem of gene flow rather than merely gene emergence. Among the three principal mechanisms of horizontal gene transfer (HGT); transformation, transduction, and conjugation, plasmid-mediated conjugation occupies a uniquely central and powerful role in disseminating clinically critical resistance determinants across taxonomic and ecological boundaries (Arredondo-Alonso et al., 2025; Koh et al., 2025; Sørensen et al., 2005; Thomas and Nielsen, 2005). Conjugation is a highly efficient, active process that transfers large DNA payloads over considerable phylogenetic distances. A single donor cell carrying a broad-host-range plasmid can, within hours, establish resistance in diverse bacterial lineages across human, animal, and environmental compartments, converting entire susceptible populations (Lopatkin et al., 2016).

**Figure 1:**
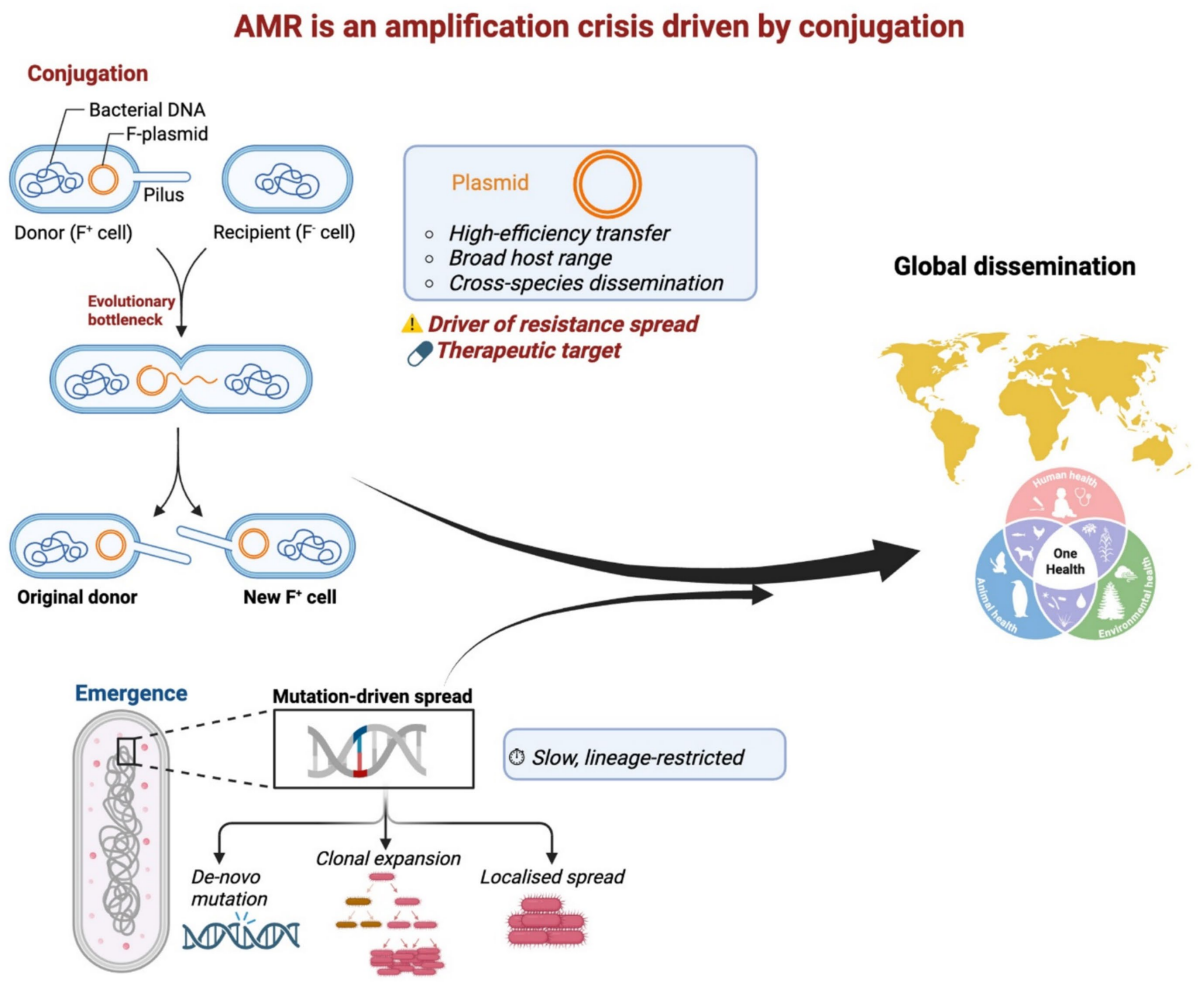
Antimicrobial resistance as an amplification crisis driven by conjugation. Antimicrobial resistance emerges through mutation and mobilization but achieves global dissemination through amplification. Mutation-driven resistance is slow and lineage-restricted, producing localized clonal expansion. In contrast, plasmid-mediated conjugation functions as a high-efficiency amplification mechanism, enabling rapid transfer of resistance determinants [e.g., blaNDM, mcr-1, tet(X4)] across diverse bacterial hosts. This process drives global dissemination within interconnected human, animal, and environmental reservoirs. Conjugation therefore acts as both the dominant amplifier of antimicrobial resistance and a critical, therapeutically targetable evolutionary bottleneck.

This review argues that conjugation is not simply a passive conduit for resistance genes but the dominant amplifier of plasmid-mediated antimicrobial resistance, and consequently represents a critical and therapeutically targetable evolutionary bottleneck. This duality; conjugation as both the core problem and a promising opportunity, frames this entire analysis. The central conceptual move is to elevate conjugation from a mechanism to be described to an intervention node to be exploited. We synthesize evidence across multiple scales: from the molecular architecture of Type IV Secretion Systems to the population dynamics of plasmid dissemination, from controlled laboratory assays to One Health surveillance data, and from evolutionary theory to emerging therapeutic strategies. A conceptual diagram of this is offered in Figure 1.

## 2 Reframing antimicrobial resistance as an amplification crisis

### 2.1 The limits of mutation-centric explanations

Mutation and clonal expansion remain foundational to understanding antimicrobial resistance. Chromosomal mutations conferring resistance to fluoroquinolones, rifampicin, and daptomycin arise spontaneously during replication and are enriched under antibiotic selection, driving local outbreaks (Woodford and Ellington, 2007). However, mutation-centric models encounter clear explanatory limits when confronted with the global resistance landscape. Multiple genomic, epidemiological, and evolutionary studies converge on the conclusion that conjugative plasmids disproportionately mediate the dissemination of clinically important resistance genes, frequently driving cross-species and global spread beyond what can be explained by clonal expansion alone (Arredondo-Alonso et al., 2025; Koh et al., 2025; Castañeda-Barba et al., 2024; Zamudio et al., 2024; Coluzzi and Rocha, 2025; Yang et al., 2024; Lipworth et al., 2024; Lian et al., 2025).

First, the extensive phylogenetic distribution of identical resistance genes exceeds what can be generated by clonal diversification within relevant timescales. The *blaNDM-1* carbapenemase gene has been identified in over 50 bacterial species spanning multiple phyla, including *Enterobacteriaceae*, *Acinetobacter*, *Pseudomonas*, and *Vibrio* (Johnson and Woodford, 2013; Shaw et al., 2025). Such breadth cannot be explained by mutation and vertical inheritance alone and instead implicates HGT as the dominant process. Consistent with this, evolutionary analyses show that plasmids are among the fastest-evolving genetic elements in bacteria and exhibit exceptionally broad host ranges, enabling rapid cross-species dissemination of resistance determinants (Coluzzi and Rocha, 2025). Experimental evidence further extends this paradigm, demonstrating interphylum transfer of clinically relevant resistance plasmids within complex microbial communities, thereby challenging traditional host-range constraints (Yang et al., 2024).

Second, resistance genes are frequently embedded within diverse mobile genetic elements (MGEs), including plasmids, transposons, integrons, insertion sequences, integrative conjugative elements(ICEs), and phage-plasmid hybrid systems, which collectively facilitate their capture, rearrangement, chromosomal integration, recombination, and dissemination (Frost et al., 2005; Rozwandowicz et al., 2018). These elements frequently operate as interconnected and nested mobility systems in which integrons capture resistance gene cassettes, transposons and insertion sequences mediate genomic rearrangement and mobilization, ICEs facilitate chromosomal transfer, and conjugative plasmids amplify dissemination across bacterial populations and species (Frost et al., 2005; Smillie et al., 2010). Among these, conjugative plasmids occupy a particularly important epidemiological role because they provide the transfer machinery necessary for horizontal movement across bacterial populations and species boundaries (Smillie et al., 2010; Redondo-Salvo et al., 2020). In community-based surveillance studies, diverse commensal *Escherichia coli* lineages have been shown to share near-identical IncF plasmids carrying common resistance determinants, indicating that in addition to clonal expansion, plasmid transmission significantly drives dissemination in community settings (Salinas et al., 2019). Similarly, in neonatal sepsis cohorts, *blaNDM-5*-producing *E. coli* isolates from distinct phylogenetic backgrounds carried highly conserved IncX3 plasmids, further highlighting the decoupling of gene spread from host lineage (Manyahi et al., 2022). Additionally, recent genomic analyses reveal a convergence of antibiotic resistance and virulence determinants on shared plasmid backbones, indicating that plasmids function as multifunctional adaptive platforms that enhance both survival and transmissibility across bacterial populations (Lian et al., 2025).

Third, the temporal dynamics of resistance emergence and dissemination often align more closely with mobilization and horizontal spread than with repeated independent mutational emergence alone. For example, although the *mcr-1* gene was first formally described in 2015, retrospective analyses subsequently identified related determinants in isolates dating back to the 1980s (Wang et al., 2018; Shen et al., 2018). This pattern likely reflects both the historical under detection of resistance determinants prior to widespread genomic surveillance and the rapid dissemination of pre-existing resistance genes following their mobilization onto successful plasmid backbones. Together, these observations highlight the important contribution of horizontal gene transfer to the global amplification of antimicrobial resistance.

### 2.2 Defining amplification velocity

If emergence represents the origin of resistance mutations or the mobilization of resistance genes onto mobile elements, amplification represents the population-level process governing dissemination velocity. Amplification velocity can be conceptualized as the rate at which a resistance determinant expands its host range and geographic distribution, shaped by the intrinsic transfer efficiency of mobile elements, ecological connectivity between microbial populations, permissive host range and selective pressures favoring plasmid carriage (Smillie et al., 2010).

Conjugation dominates amplification because it uniquely integrates high transfer efficiency with broad host range. Transformation depends on environmental DNA and recipient competence, while transduction is constrained by bacteriophage host range and packaging limitations. In contrast, conjugation actively delivers DNA through a dedicated secretion system capable of functioning across diverse recipient species, often with minimal phylogenetic constraint (Sørensen et al., 2005; Frost et al., 2005). The evolutionary properties of plasmids including rapid evolution, modular architecture, and broad host adaptability position them as key determinants of amplification velocity, enabling the transition from localized emergence to global dissemination (Coluzzi and Rocha, 2025). This capacity is further amplified by their ability to traverse phylogenetic boundaries and circulate within environmental reservoirs, reinforcing conjugation as a mechanism capable of large-scale resistance propagation (Yang et al., 2024). In this framework, plasmids operate as mobile, evolvable platforms that couple resistance emergence to rapid ecological and evolutionary spread, consistent with an amplification-driven model of antimicrobial resistance (Castañeda-Barba et al., 2024).

### 2.3 Epidemiological evidence for conjugation dominance

Quantitative assessment of conjugation’s dominance requires examining the association between clinically critical resistance genes and plasmid backbones capable of autonomous transfer. Systematic analyses of public genome databases reveal that genes conferring resistance to last-resort antibiotics are disproportionately located on conjugative or mobilizable plasmids (Rozwandowicz et al., 2018; Carattoli, 2013).

Carbapenemase genes of the *blaNDM*, *blaKPC*, and *blaOXA-48* families are overwhelmingly plasmid-encoded. Among 5,000 + *blaNDM*-harboring genomes in the NCBI database, over 95% carry the gene on plasmids assignable to known incompatibility groups, predominantly IncF, IncX3, and IncC (Acman et al., 2022). Similarly, the *mcr* gene family shows strong associations with IncI2, IncHI2, and IncX4 plasmid backbones, all capable of conjugation (Lu et al., 2020; Karpenko et al., 2024; Feng et al., 2019). The recently emerged *tet(X4)* tigecycline resistance gene has been identified on IncI1, IncF, and IncHI1 plasmids in diverse bacterial hosts, demonstrating the rapid capture of a novel resistance mechanism by pre-existing, highly successful plasmid scaffolds (Mansoor et al., 2024; Phuadraksa et al., 2025).

Comparative genomics and outbreak investigations increasingly demonstrate that clinically significant resistance dissemination is frequently driven by plasmid transmission rather than clonal expansion alone. Whole-genome sequencing analyses of carbapenemase-producing *Enterobacterales* revealed extensive dissemination of conserved *blaKPC-2*- and *blaNDM-1*-carrying plasmids across genetically distinct strains and multiple bacterial species, patterns that conventional clone-focused surveillance approaches had failed to detect (Marimuthu et al., 2022). In nationwide analyses, dominant plasmid clusters accounted for the majority of transmission events through horizontal transfer (~60%), whereas clonal inheritance contributed comparatively less (~40%) (Koh et al., 2025). Importantly, clonal spread was largely confined to individual healthcare institutions, while plasmid-mediated transmission occurred across multiple hospitals, species, and phylogenetic backgrounds, indicating that conjugative plasmids function as major drivers of regional dissemination (Koh et al., 2025; Marimuthu et al., 2022). Similar findings were reported during a multispecies carbapenemase-producing *Enterobacterales* outbreak in a pediatric ward in Tokyo, where identical IncM1 plasmids carrying *blaIMP-1* disseminated across multiple bacterial species, patients, and sink-associated environmental reservoirs despite substantial strain diversity (Tsukada et al., 2024). The persistence of these plasmids across both clinical and environmental compartments demonstrated that horizontal plasmid transfer, rather than expansion of a single epidemic clone, was the dominant mechanism sustaining the outbreak (Koh et al., 2025; Marimuthu et al., 2022; Tsukada et al., 2024; Bolourchi et al., 2021; Li et al., 2024). Nonetheless, it is important to highlight that plasmid-mediated dissemination and clonal expansion are not mutually exclusive processes, but frequently interact synergistically, with successful epidemic clones often serving as highly permissive hosts for transmissible resistance plasmids.

At a global scale, longitudinal, population-level, and high-resolution plasmidome analyses consistently reinforce the interconnected roles of conjugation and clonal expansion in antimicrobial resistance dissemination. For example, a limited number of conserved epidemic plasmid backbones are repeatedly associated with the emergence, expansion, and persistence of major Extraintestinal pathogenic *Escherichia coli* (ExPEC) lineages across diverse geographic and phylogenetic contexts (Arredondo-Alonso et al., 2025; Koh et al., 2025; Zamudio et al., 2024). Studies by Mathers et al. and Pitout et al. further demonstrate that epidemic IncF plasmids have played pivotal roles in the global success of major MDR ExPEC clones such as ST131 and ST410 through long-term plasmid–host compatibility, compensatory adaptation, and stable lineage–plasmid coevolution (Mathers et al., 2015; Pitout and Chen, 2023). In particular, the IncF/*CTX-M-15*/ST131-C2 combination represents one of the most successful clone–plasmid partnerships associated with global antimicrobial resistance dissemination (Pitout and Chen, 2023). Certain epidemic lineages may therefore function as highly permissive hosts that buffer plasmid-associated fitness costs and promote long-term maintenance of transmissible resistance determinants. Complete plasmid assemblies from bloodstream infections further demonstrate that these transmissible backbones mediate cross-species dissemination of resistance determinants in clinical settings, frequently carrying multidrug-resistance cargo and circulating across distinct bacterial hosts (Lipworth et al., 2024). Together, these findings suggest that the regional and global expansion of epidemic resistant clones may often reflect synergistic interactions between advantageous chromosomal backgrounds and evolutionarily optimized plasmid backbones rather than purely clonal dissemination alone.

**Figure 2:**
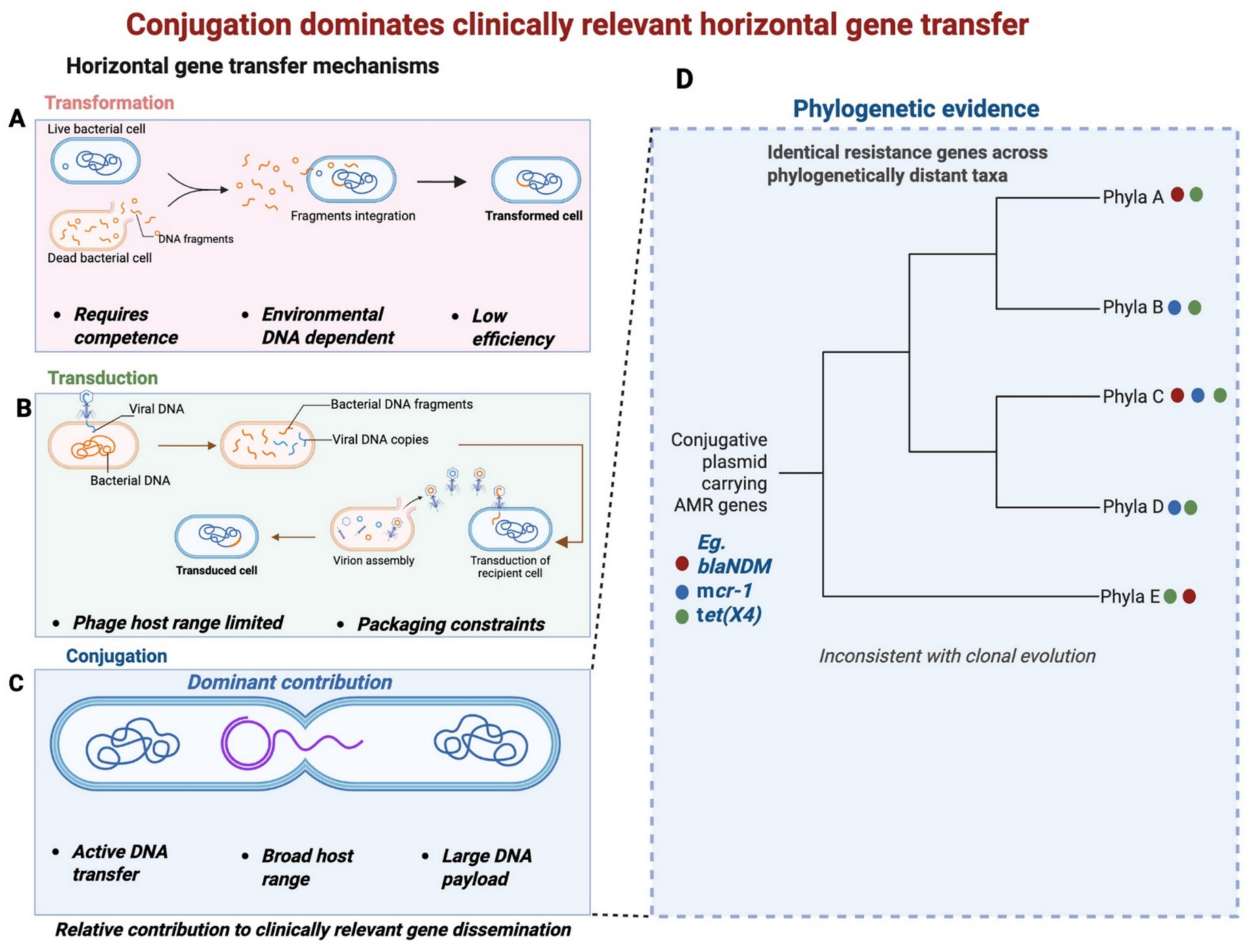
Conjugation dominates clinically relevant horizontal gene transfer. **(A)** Transformation, in which extracellular DNA released from dead bacterial cells is taken up and integrated by competent recipient bacteria. This process is constrained by environmental DNA availability and recipient competence. **(B)** Transduction, in which bacteriophages transfer bacterial DNA between cells. Transduction is limited by phage host range and DNA packaging constraints. **(C)** Conjugation, which enables direct cell-to-cell transfer of DNA via conjugative plasmids and is characterized by active transfer, broad host range, and the ability to mobilize large DNA cargo. **(D)** Phylogenetic evidence supporting conjugation-mediated dissemination of antimicrobial resistance determinants. Identical resistance genes, including *blaNDM*, *mcr-1*, and *tet(X4)*, are observed across phylogenetically distant bacterial taxa, a pattern inconsistent with clonal evolution alone and indicative of horizontal transfer mediated by conjugative plasmids.

This evolutionary relationship also generates an important challenge known as the plasmid paradox. As discussed by Brockhurst and Harrison, although plasmids often impose fitness costs, beneficial plasmid-borne resistance or virulence determinants may subsequently become integrated into the bacterial chromosome, stabilizing adaptive traits while reducing selective pressure for long-term plasmid maintenance (Brockhurst and Harrison, 2022). This is complimented by evidence showing that chromosomal integration of resistance genes previously carried on plasmids can promote plasmid loss while permanently enriching successful bacterial lineages with resistance cargo (Zongo et al., 2024). However, plasmid loss is not inevitable as compensatory evolution, reduced fitness burden, addiction systems, and partition systems can stabilize plasmid persistence and generate mutually beneficial plasmid–host relationships (Arredondo-Alonso et al., 2025; San Millan, 2018). This is particularly evident in the emergence of hybrid resistance–virulence “super plasmids,” including ColV-like plasmids in ExPEC and virulence-resistance plasmids in high-risk *Klebsiella pneumoniae* lineages, which simultaneously enhance pathogenicity, ecological fitness, persistence, and antimicrobial resistance. Such plasmid-mediated advantages may partly underpin the remarkable success and global dissemination of certain epidemic clones, suggesting that apparent clonal dominance may, in many cases, conceal a deeper plasmid-driven evolutionary architecture (Figure 2).

## 3 Why conjugation dominates clinically relevant gene flow

Conjugation is uniquely positioned to dominate clinically relevant gene flow due to a combination of structural efficiency, ecological flexibility, and evolutionary scalability. The Type IV secretion system (T4SS) that mediates conjugation is a highly specialized trans-envelope machine capable of delivering DNA directly into recipient cells, bypassing extracellular exposure and thereby avoiding degradation (Costa et al., 2021; Ilangovan et al., 2015). DNA is transferred as a protein-associated complex, with the relaxase covalently bound to the single-stranded substrate, ensuring stability during transfer and facilitating rapid reconstitution in the recipient (Williams et al., 2025; de la Cruz et al., 2010).

Beyond transfer efficiency, conjugative plasmids are intrinsically self-establishing. In contrast to transformation or transduction, which require compatibility with host machinery, conjugative elements encode the full repertoire required for replication, partitioning, and maintenance across diverse bacterial backgrounds (Thomas, 2000). Broad-host-range plasmids, including those of the IncP, IncN and IncA/C groups, can therefore establish in phylogenetically distant hosts, enabling cross-species dissemination of resistance determinants (Jain and Srivastava, 2013; Suzuki, 2019).

A defining feature of conjugation is its hierarchical mobility. In addition to transfering themselves, conjugative plasmids can also mobilize non-conjugative or apparently non-self-transmissible plasmids in trans, allowing a limited number of transfer-competent elements to disseminate diverse resistance determinants across bacterial communities (Redondo-Salvo et al., 2020). Consequently, clinically important outbreaks may involve cooperative plasmid networks in which mobilizable resistance plasmids exploit shared conjugation machinery provided by co-resident helper plasmids (Frost et al., 2005; Smillie et al., 2010). This networked architecture further amplifies resistance dissemination beyond the boundaries of autonomous conjugative elements (Rozwandowicz et al., 2018; Redondo-Salvo et al., 2020).

These mechanistic advantages are reflected in the epidemiology of clinically dominant plasmid families. Rather than being randomly distributed, resistance genes are preferentially associated with plasmid backbones that balance host range, stability, and transfer efficiency (Koh et al., 2025). IncF plasmids dominate ESBL dissemination within Enterobacteriaceae, reflecting their capacity for gene accumulation and host adaptation (Rozwandowicz et al., 2018; Salinas et al., 2019). In contrast, IncA/C, IncX and IncI plasmids exhibit broader host ranges and high transfer efficiencies, enabling rapid interspecies dissemination of carbapenemases and colistin resistance genes (Shaw et al., 2025; Manyahi et al., 2022; Lu et al., 2020; Fu, 2025)^.^ Whereas IncH1 plasmids are larger low copy, thermoregulated plasmids known to express surface proteins containing antigenic immunoglobulin like domains. A finding with direct therapeutic implications (Prieto, 2024).

Together, these observations indicate that the clinical success of resistance plasmids is not determined solely by the genes they carry, but by the transmission architecture of the plasmid backbone. Conjugation therefore functions not merely as a mechanism of transfer, but as an integrated system that couples efficient DNA delivery, cross-host establishment, and network-level amplification features that collectively underpin its dominant role in the dissemination of antimicrobial resistance (Table 1).

**Table 1:** Clinically significant plasmid families associated with antimicrobial resistance genes.

Plasmid family	Host range	Key resistance genes	Associated bacterial hosts	Clinical significance	Citations
IncF	Narrow (Enterobacteriaceae)	blaCTX-M, blaNDM, blaTEM, blaSHV, qnr, aac(6′)-Ib	*E. coli*, *K. pneumoniae*, *Salmonella.*	Most prevalent in clinical ESBL isolates; modular structure facilitates gene accretion	Frost et al. (2005), Rozwandowicz et al. (2018), Manyahi et al. (2022)
IncI (IncI1, IncI2)	Narrow to intermediate	mcr-1, mcr-4, blaCTX-M, blaCMY	*E. coli*, *K. pneumoniae*, *Salmonella*	Dominant vectors for colistin resistance; common in animal and environmental isolates	Acman et al. (2022), Karpenko et al. (2024), Mansoor et al. (2024)
IncHI (IncHI1, IncHI2)	Broad (Enterobacteriaceae)	blaCTX-M, blaNDM, mcr, qnr, flor, sul, tet(X4)	*Salmonella*, *E. coli*, *K. pneumoniae*	Large thermoregulated plasmids; associated with MDR in enteric pathogens; surface Big domains antigenic	Ilangovan et al. (2015), Williams et al. (2025), Frost and Koraimann (2010)
IncA/C	Broad (Gram-negative)	blaNDM, blaIMP, blaCMY, floR, sul, tet	*E. coli*, *Salmonella*, *Vibrio*, *Klebsiella*	Key vectors for carbapenemases; megaplasmids carry multiple resistance determinants	Shaw et al. (2025), San Millan (2018)
IncX (IncX3, IncX4)	Narrow to intermediate	blaNDM-5, mcr-1, qnr	*E. coli*, *K. pneumoniae*	Small size, high conjugation efficiency; rapid dissemination	Rozwandowicz et al. (2018), Carattoli (2013)
IncP	Very broad (Gram-negative)	Various (often multi-resistance)	Diverse environmental and clinical bacteria	Promiscuous transfer across phyla; potential delivery vectors for therapeutic constructs	Zongo et al. (2024)

## 4 Evolutionary economics of conjugative transfer

Conjugative transfer is not constitutive but economically constrained. Although often described as energetically costly, quantitative estimates indicate that the direct mechanical ATP expenditure, such as that associated with helicase activity and type IV secretion (~10^5^–10^6^ ATP per event) constitutes only a minor fraction of whole-cell energy turnover (Pyle, 2008; Hu et al., 2019; Macé et al., 2022; Cabezón et al., 2023). Instead, the dominant physiological burden arises from transient perturbations in proteome allocation, translation, and nucleotide flux during plasmid establishment (Couturier et al., 2023; San Millan and MacLean, 2017; Roberts et al., 2021; Tamminen et al., 2012; Baltrus, 2013).

Following transfer, entry of a ~ 100-kb single-stranded plasmid can transiently sequester a substantial fraction of the cellular single-stranded DNA-binding protein (SSB) pool, imposing a protein-allocation constraint on DNA metabolism (Couturier et al., 2023; Spenkelink et al., 2019; Zhao, 2019). Concurrent activation of single-stranded promoters initiates early gene expression, increasing translational demand, followed by rapid nucleotide incorporation (~10^5^ high-energy bonds) during complementary strand synthesis (Dodd et al., 2020) and replication-driven copy-number recovery. The largest biosynthetic investment arises during maturation, where expression of plasmid-encoded functions may require synthesis on the order of 10^6^–10^7^ amino acids (Couturier et al., 2023; Soufi et al., 2015; Tiessen et al., 2012). These temporally concentrated demands suggest that transient constraints on protein availability and translational capacity, rather than absolute ATP limitation, define a key physiological bottleneck during plasmid establishment.

These costs translate into measurable fitness effects, with plasmids often reducing host growth by 10–30%, particularly in novel hosts (San Millan and MacLean, 2017). However, compensatory evolution in both plasmid and chromosome can rapidly ameliorate these costs, stabilizing plasmid persistence even in the absence of antibiotic selection (Zwanzig, 2019; Harrison and Brockhurst, 2012; Storey et al., 2022). Importantly, emerging evidence indicates that this persistence is not solely a consequence of cost compensation but also reflects the acquisition of auxiliary fitness benefits, including the co-localization of virulence and resistance determinants on shared plasmid backbones, which can enhance host colonization and ecological competitiveness (Lian et al., 2025). This adaptive buffering creates a transient and context-dependent window during early establishment in which plasmid–host associations remain vulnerable to perturbation, representing a potential point of therapeutic intervention.

Plasmids further reinforce persistence through addiction systems (toxin–antitoxin modules), which eliminate plasmid-free segregants, a phenomenon inducible under antibiotic stress, thereby coupling selection pressure to plasmid stabilization (Storey et al., 2022; Burrus and Waldor, 2004; Qi et al., 2021; Botelho et al., 2023). Conjugation itself is tightly regulated, reflecting an economic logic (Williams et al., 2025; Wang and Joffré, 2025; Ramiro-Martínez et al., 2025); quorum sensing, stress responses such as SOS induction, and metabolic state collectively gate transfer, ensuring that costly conjugation is deployed only under favorable conditions (Qi et al., 2021; Piper and Farrand, 2000; Frost and Koraimann, 2010; Beaber et al., 2004). This regulatory coupling creates exploitable nodes where disruption of signaling or stress-response pathways may decouple transfer activation from permissive ecological contexts.

**Figure 3:**
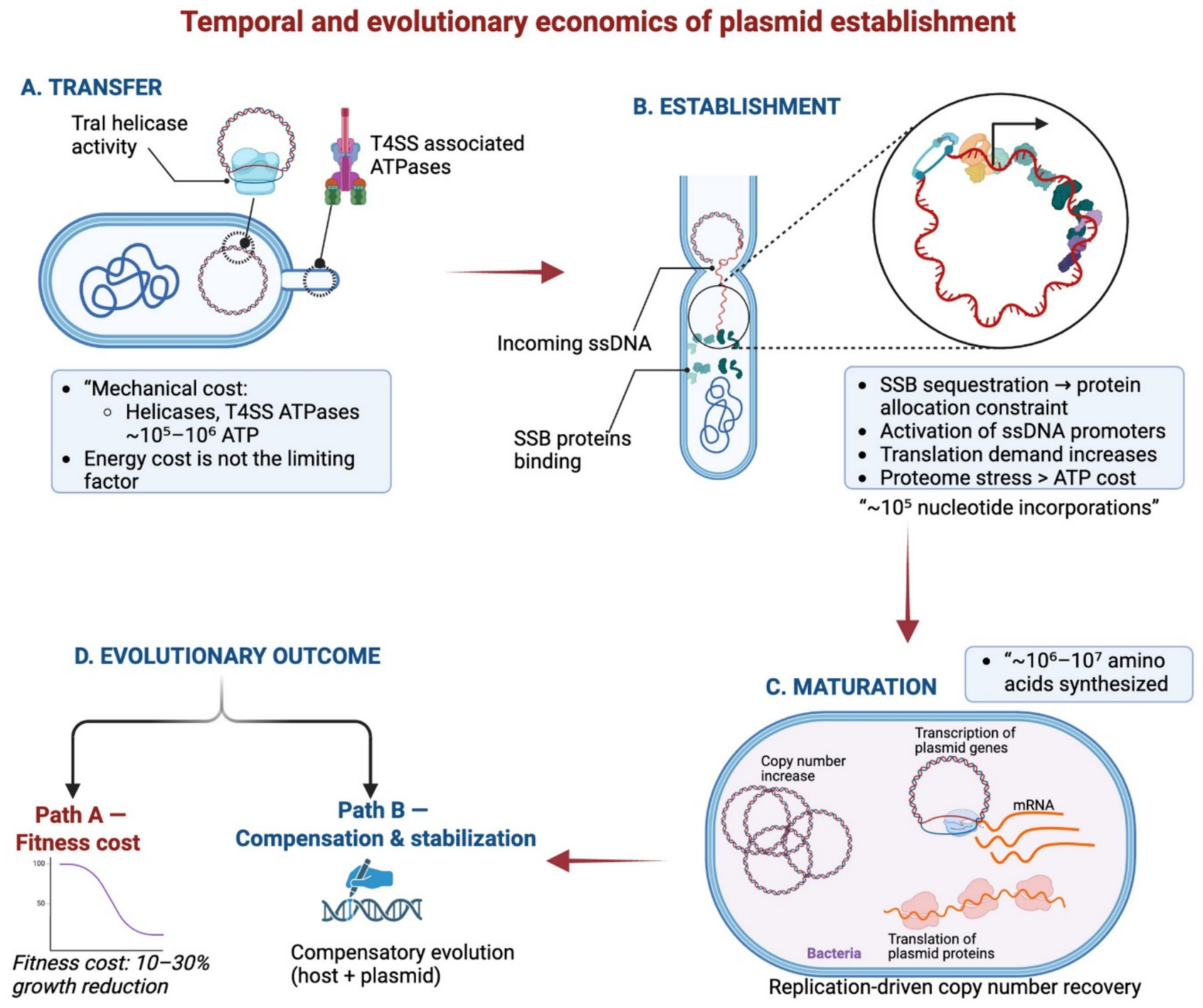
Temporal and evolutionary economics of plasmid establishment. **(A)** Transfer. Conjugative DNA transfer via the type IV secretion system incurs modest energetic cost relative to total cellular metabolism. **(B)** Establishment. Incoming single-stranded plasmid DNA imposes transient constraints on protein allocation and nucleotide flux, generating competition for cellular resources and defining an early physiological bottleneck. **(C)** Maturation. Plasmid stabilization is associated with sustained transcription, replication-driven copy number recovery, and large-scale protein synthesis, representing peak biosynthetic demand. **(D)** Evolutionary outcomes. Initial fitness costs are mitigated through compensatory evolution, while additional adaptive benefits can promote long-term plasmid persistence. Conjugation is regulated by environmental and physiological signals, and long-term plasmid success reflects trade-offs between biosynthetic burden, host fitness, and transmissibility.

At longer evolutionary timescales, these constraints drive structural optimization. Dominant epidemic plasmids exhibit conserved, size-restricted architectures, whereas expanded variants are rare, indicating selection for streamlined backbones under biosynthetic limitation (Koh et al., 2025; Matthews et al., 2025). Under conditions favoring horizontal transmission, further streamlining through deletion of costly regions can enhance transfer efficiency, revealing an intrinsic trade-off between resistance cargo and transmissibility that may be leveraged to selectively disadvantage resistance-bearing plasmids (Matthews et al., 2025; Lopatkin et al., 2017) (Figure 3).

Taken together, conjugation is best understood as a temporally partitioned and tightly regulated metabolic program shaped by trade-offs between transfer efficiency, host fitness, plasmid copy number, and biosynthetic capacity. These constraints govern plasmid persistence and also defines discrete physiological and regulatory vulnerabilities that can be exploited to limit the amplification of antimicrobial resistance (Table 2).

**Table 2:** Therapeutic strategies targeting conjugation as an evolutionary bottleneck.

Process stage	Biophysical basis of estimate	Assumptions and scaling logic	Estimated magnitude	Key references
DNA unwinding (donor)	SF1 helicase (TraI) translocation	~1 ATP hydrolysed per nucleotide unwound; 100–108 kb plasmid length	~10^5^ ATP	Pyle (2008)
DNA translocation (T4SS)	ATPases (VirB4/VirB11) driving substrate transfer	No nucleotide-resolution estimate; extrapolated from secretion systems transferring large substrates	~10^5^–10^6^ ATP	Hu et al. (2019), Macé et al. (2022), Cabezón et al. (2023)
ssDNA stabilization (recipient)	SSB binding to incoming ssDNA	~35 nt occluded per SSB tetramer [(SSB)₃₅ mode]; ~100 kb ssDNA → ~ 3,000 tetramers; cellular pool ~2,000–4,000	Large fraction of SSB pool engaged (protein allocation constraint)	Couturier et al. (2023), Spenkelink et al. (2019), Zhao (2019)
Complementary strand synthesis	DNA polymerization chemistry	1 nucleotide triphosphate incorporated per base; ~100–108 kb strand synthesized	~10^5^ ATP-equivalent bonds (minimum)	Dodd et al. (2020)
Copy-number recovery	Plasmid replication after establishment	Additional rounds of replication (ΔC copies); linear scaling with plasmid size	~10^5^–10^6^ ATP equivalents	San Millan and MacLean (2017), Ramiro-Martínez et al. (2025)
Early gene expression	Translation of leading-region genes	~4 high-energy bonds per amino acid (ATP + GTP equivalents); early burst expression	~10^6^ ATP equivalents (order of magnitude)	Couturier et al. (2023)
Maturation (transfer competence)	Synthesis of conjugation machinery (Tra proteins)	Scaling of protein number (~30–40), size (~300–500 aa) and expression (~10^2^–10^3^ copies) required for donor competence	~10^6^–10^7^ amino acids (~4 × 10^6^–10^7^ ATP equivalents)	Couturier et al. (2023), Soufi et al. (2015)
Cellular context scaling	Whole-cell ATP turnover	Rapidly growing bacteria: ~10^9^ ATP per minute (order of magnitude)	Mechanical costs <1% of minute-scale ATP flux	San Millan and MacLean (2017)

Estimates are derived from established biochemical parameters and scaled to a ~ 100–108 kb conjugative plasmid. Mechanical ATP costs associated with helicase activity and secretion are calculated from per-nucleotide or system-level energetic requirements, whereas biosynthetic costs are inferred from nucleotide incorporation and protein synthesis stoichiometry. Protein allocation constraints are estimated from SSB binding stoichiometry relative to cellular abundance. Values represent order-of-magnitude approximations intended to compare relative energetic burdens across stages rather than precise measurements. Collectively, these estimates show that conjugation imposes limited direct ATP demand but substantial, temporally concentrated constraints on proteome allocation, translation, and nucleotide availability.

## 5 Stress landscapes and the acceleration of gene flow

### 5.1 Anthropogenic contexts as amplifiers

Anthropogenic environments generate pervasive sublethal stress landscapes that actively accelerate horizontal gene transfer. Sub-MICs of antibiotics, commonly encountered in clinical and environmental settings, not only select for resistant bacteria but also increase conjugation frequencies through induction of oxidative stress, activation of the SOS response, and modulation of donor–recipient physiology (Lopatkin et al., 2016; Yu et al., 2021; Zhang et al., 2025). This establishes a positive feedback loop in which antibiotic exposure simultaneously selects for and propagates resistance.

Importantly, this phenomenon extends beyond antibiotics. Diverse environmental stressors including microplastics, bisphenol compounds, nonnutritive sweeteners, heavy metals, and cigarette smoke constituents converge on shared physiological pathways that enhance conjugative transfer. These include increased reactive oxygen species production, membrane perturbation, and upregulation of transfer-associated genes, collectively creating transfer-permissive cellular states (Yu et al., 2021; Figueiredo et al., 2019; Milani, 2024; Yang et al., 2025; Yang et al., 2024; Yang et al., 2025; He et al., 2023; Fang et al., 2025). Rather than acting independently, such stressors frequently co-occur, particularly in wastewater and polluted ecosystems, generating synergistic conditions that amplify plasmid dissemination.

Within this landscape, phytochemicals introduce a globally relevant and underappreciated dimension. In low- and middle-income countries, where the WHO estimates that traditional herbal medicine constitutes a primary component of healthcare (Khan et al., 2025), plant-derived compounds may represent a pervasive chemical environment shaping plasmid dissemination. Emerging evidence indicates that these effects are bidirectional: subinhibitory exposure to polyherbal formulations and certain phytochemicals can enhance conjugative transfer, raising the possibility that routine therapeutic use may inadvertently contribute to resistance amplification (Wang et al., 2024; Kwapong et al., 2020). Conversely, other phytochemicals suppress plasmid transfer across diverse systems, including inhibition of gene exchange between multidrug-resistant *Salmonella* and commensal *Escherichia coli* (Vinayamohan, n.d.), and modulation of transfer in defined plasmid models (Kwapong et al., 2020; Bañuelos-Vazquez et al., 2020). These findings position phytochemical exposure as a context-dependent regulator of conjugation and suggest that widespread herbal medicine use may represent an underrecognized contributor to resistance dynamics in high-burden settings.

Within a One Health framework, these stress landscapes intersect with highly connected ecological networks that facilitate plasmid circulation across human, animal, and environmental reservoirs. Asymptomatic human carriage, agricultural systems, companion animals, and contaminated water environments collectively sustain a continuous exchange network in which conjugative plasmids circulate, recombine, and re-enter clinical settings (Karpenko et al., 2024; Pungpian et al., 2021; Habib et al., 2026; Mahmoodi et al., 2020; Debergh et al., 2022). Emerging hotspots, including wastewater systems and anthropogenically influenced wildlife niches, further intensify these dynamics by coupling high microbial density with sustained environmental stress (Mansoor et al., 2024; Phuadraksa et al., 2025).

**Figure 4:**
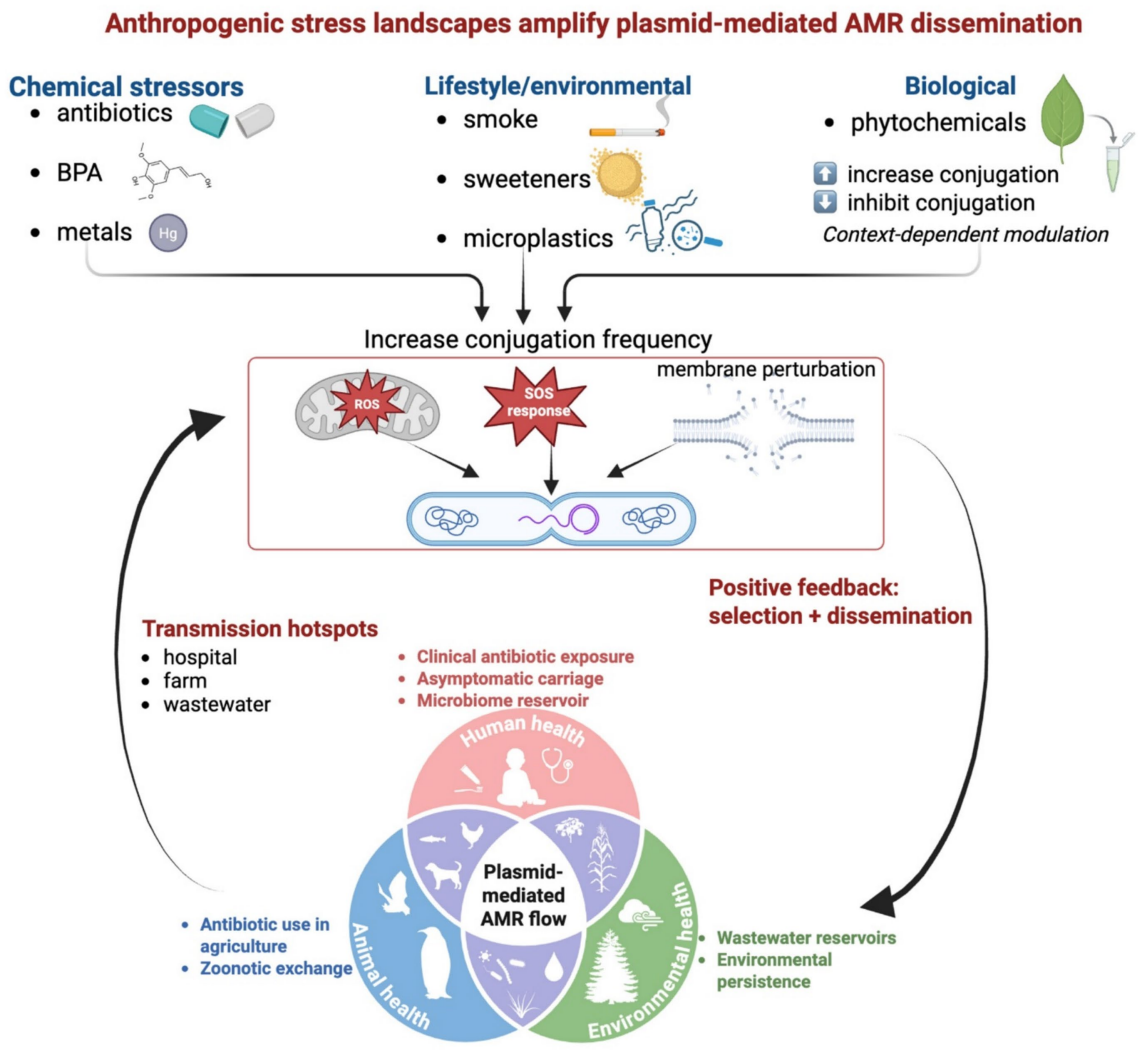
Anthropogenic stress landscapes amplify plasmid-mediated antimicrobial resistance dissemination. Diverse anthropogenic stressors, including sublethal antibiotics, environmental pollutants, and phytochemicals, induce physiological responses such as oxidative stress, SOS activation, and membrane perturbation that enhance conjugative transfer. These stress-induced cellular states increase plasmid exchange between bacteria, driving the amplification of resistance genes. Within interconnected One Health systems, plasmids circulate between human, animal, and environmental reservoirs, with transmission hotspots such as hospitals, farms, and wastewater systems intensifying dissemination. Phytochemicals introduce a context-dependent dimension, with both enhancing and inhibitory effects on conjugation. Together, these interactions establish a positive feedback loop linking environmental exposure, selection, and horizontal gene transfer, thereby accelerating the spread of antimicrobial resistance.

Together, these findings indicate that conjugation is not merely a baseline biological process but is actively amplified by anthropogenic stress regimes, transforming environmental exposure landscapes into engines of resistance dissemination (Figure 4).

## 6 Conjugation as an evolutionary bottleneck

If resistance emergence reflects mutation at the level of individual cells, conjugation operates as the population-level amplifier that governs dissemination velocity. This reframing elevates conjugation from a mechanistic process to a strategic intervention node. In transmission networks, spread is constrained by rate-limiting steps; for plasmid-mediated resistance, conjugation frequently constitutes a dominant rate-limiting step in dissemination. The relationship is such that reductions in conjugation efficiency directly dampen amplification velocity.

Epidemiological and theoretical models support this framework. The plasmid reproductive number (R₀), analogous to that of infectious pathogens, determines whether a plasmid can invade and persist within a population (Roberts et al., 2021). Mathematical models of plasmid dynamics consistently identify conjugation rate as a dominant parameter governing plasmid persistence, with even modest reductions sufficient to shift systems from stable maintenance to extinction (Hernández-Beltrán et al., 2021; Yang et al., 2026). More broadly, plasmid dynamics emerge from the interaction between transfer efficiency, fitness cost, and segregational stability, reinforcing the view that resistance dissemination is governed by quantitative trade-offs rather than mutation alone (Zwanzig, 2019; Wang and Joffré, 2025; Hernández-Beltrán et al., 2021; Yang et al., 2026).

At the intracellular level, these dynamics are further shaped by constraints on plasmid replication and gene dosage. Plasmid copy number follows conserved quantitative rules and is dynamically regulated to balance resistance expression against metabolic burden (Wang and Joffré, 2025; Ramiro-Martínez et al., 2025; Yang et al., 2026), linking intracellular optimization to population-level spread. Because copy number influences both plasmid persistence and the probability of successful transfer, these constraints couple cellular resource allocation directly to amplification dynamics.

However, the magnitude of this bottleneck is modulated by ecological context, including population structure, host density, microbiome composition, and environmental connectivity, which together shape opportunities for donor–recipient contact. In natural systems, co-infection with multiple plasmids introduces additional layers of complexity, as competitive and cooperative plasmid–plasmid interactions can modulate transfer efficiency and persistence (Siedentop et al., 2025). Despite this complexity, convergent evidence across modeling and empirical systems consistently identifies conjugation as a dominant control parameter governing dissemination.

Unlike mutation or selection, which are diffuse and difficult to control directly, conjugation represents a discrete, mechanistically defined process. This renders it uniquely amenable to targeted intervention, positioning it as a critical control point in the amplification of antimicrobial resistance.

## 7 Therapeutic targeting; feasibility and evolutionary constraints

If conjugation constitutes a rate-limiting step in resistance amplification, its disruption represents a rational and potentially high-impact therapeutic strategy. The multi-step architecture of conjugative transfer exposes multiple molecular and ecological vulnerabilities that can be exploited.

Structural studies of the type IV secretion system (T4SS) and F-pilus have revealed ATP-dependent biomechanical processes that are amenable to pharmacological targeting (Patkowski et al., 2023). Small-molecule inhibitors provide compelling evidence that the conjugation machinery is a tractable target for intervention (Cabezón et al., 2017). Compounds such as 2-hexadecynoic acid disrupt the ATPase activity of the type IV secretion system, effectively blocking plasmid transfer *in vivo*, while relaxase enzymes represent conserved molecular bottlenecks that can be inhibited across diverse plasmid families. These findings demonstrate that a limited number of core enzymatic functions underpinning conjugation can be selectively targeted to suppress horizontal gene transfer at its source (Getino et al., 2015; Getino and de la Cruz, 2018; Palencia-Gándara et al., 2021; Liu et al., 2024). Complementary approaches include nanobody-mediated interference with surface-exposed conjugation proteins (Prieto, 2024), CRISPR-based systems for sequence-specific plasmid targeting (de Souza et al., 2025; Yoon et al., 2026; Lee et al., 2025; Zhou et al., 2023), bacteriophage strategies that exploit sex pili as entry receptors (Colom et al., 2019), Phytochemicals (Kwapong et al., 2020; Vinayamohan, n.d.) and plasmid incompatibility-based displacement to eliminate resident resistance plasmids (Burrus and Waldor, 2004) (Table 3).

**Table 3:** Therapeutic strategies targeting conjugation as an evolutionary bottleneck.

Strategy	Mechanism of action	Examples	Stage of development	Advantages	Limitations/challenges	Citations
CRISPR-Cas systems	Sequence-specific cleavage or interference with plasmid DNA or RNA	CRISPR-Cas9 targeting AMR genes; endogenous CRISPR-Cas3 activation; CRISPRi	Preclinical	Programmable, sequence-specific; can cure plasmids or block acquisition	Delivery to target bacteria; off-target effects; anti-CRISPR proteins	Patkowski et al. (2023), Cabezón et al. (2017), Getino et al. (2015), Getino and de la Cruz (2018)
Small-molecule conjugation inhibitors	Inhibit T4SS ATPases, relaxases, pilus assembly, or energy metabolism	2-hexadecynoic acid; bithionol; tannic acid; acetylshikonin	Preclinical	Non-lethal (weak selection pressure); natural products may be well-tolerated	Potency, specificity; pharmacokinetics; resistance evolution	Debergh et al. (2022), Hernández-Beltrán et al. (2021), Yang et al. (2026), Siedentop et al. (2025)
Nanobodies targeting plasmid surface proteins	Bind plasmid-encoded surface proteins to block conjugation	Anti-IncHI nanobodies targeting bacterial Ig-like domains	Early research	Highly specific; target directly linked to AMR plasmid; surface-exposed	Only demonstrated for IncHI; delivery; immunogenicity	Williams et al. (2025)
Phage-delivered CRISPR	Phage particles deliver CRISPR constructs	Phagemids carrying CRISPR-Cas9	Early research	Combines phage delivery with CRISPR specificity; self-limiting	Phage host range; delivery efficiency	Palencia-Gándara et al. (2021)
Plasmid displacement vectors	Incompatibility-based displacement; addiction system neutralization	IncP-1-based curing cassettes; antitoxin-expressing vectors	Preclinical	Exploits natural plasmid biology; works without antibiotic selection	Requires selection for spread; efficiency in complex communities	Dodd et al. (2020)
Plasmid streamlining	Engineered streamlined plasmids outcompete resistance plasmids	Spontaneous deletion variants with enhanced transmission	Proof-of-concept	Intrinsic defense; could reverse resistance	Evolutionary trajectory unpredictable; safety concerns	Botelho et al. (2023)

Despite their diversity, these approaches converge on a common principle: disruption of the transfer machinery, its regulation, or its ecological accessibility. Among these strategies, small-molecule inhibitors and phage-based approaches currently appear the most translationally tractable, whereas CRISPR-based systems remain highly promising but face delivery constraints in complex microbial communities. More broadly, the existence of constrained and dynamically regulated plasmid copy number regimes suggests an additional therapeutic axis, in which perturbation of replication control could shift plasmids away from fitness-optimal states, thereby reducing resistance expression or destabilizing persistence (Wang and Joffré, 2025; Ramiro-Martínez et al., 2025).

Therapeutic targeting, however, operates within an evolutionary landscape that constrains durability. Resistance to conjugation-targeting interventions is likely to emerge, but key targets such as relaxases and ATPases perform essential and highly conserved functions within the conjugation machinery (Costa et al., 2021; de la Cruz et al., 2010; Patkowski et al., 2023), limiting the evolutionary space for escape and potentially constraining the emergence of resistance to inhibition. Because these interventions act on a bottleneck process, resistance mutations that restore transfer are likely to incur trade-offs in efficiency, stability, or host fitness, thereby constraining evolutionary escape (Zwanzig, 2019). Mathematical models further predict threshold conditions under which plasmids cannot be maintained, indicating that even partial reductions in conjugation efficiency may be sufficient to drive plasmid extinction (Hernández-Beltrán et al., 2021; Siedentop et al., 2025).

In addition, co-infection dynamics suggest that plasmid–plasmid competition could be exploited therapeutically, for example through the introduction of incompatible or engineered plasmids to displace resistance elements (Siedentop et al., 2025). Such strategies highlight the potential of leveraging intrinsic plasmid ecology rather than solely targeting individual molecular components.

**Figure 5:**
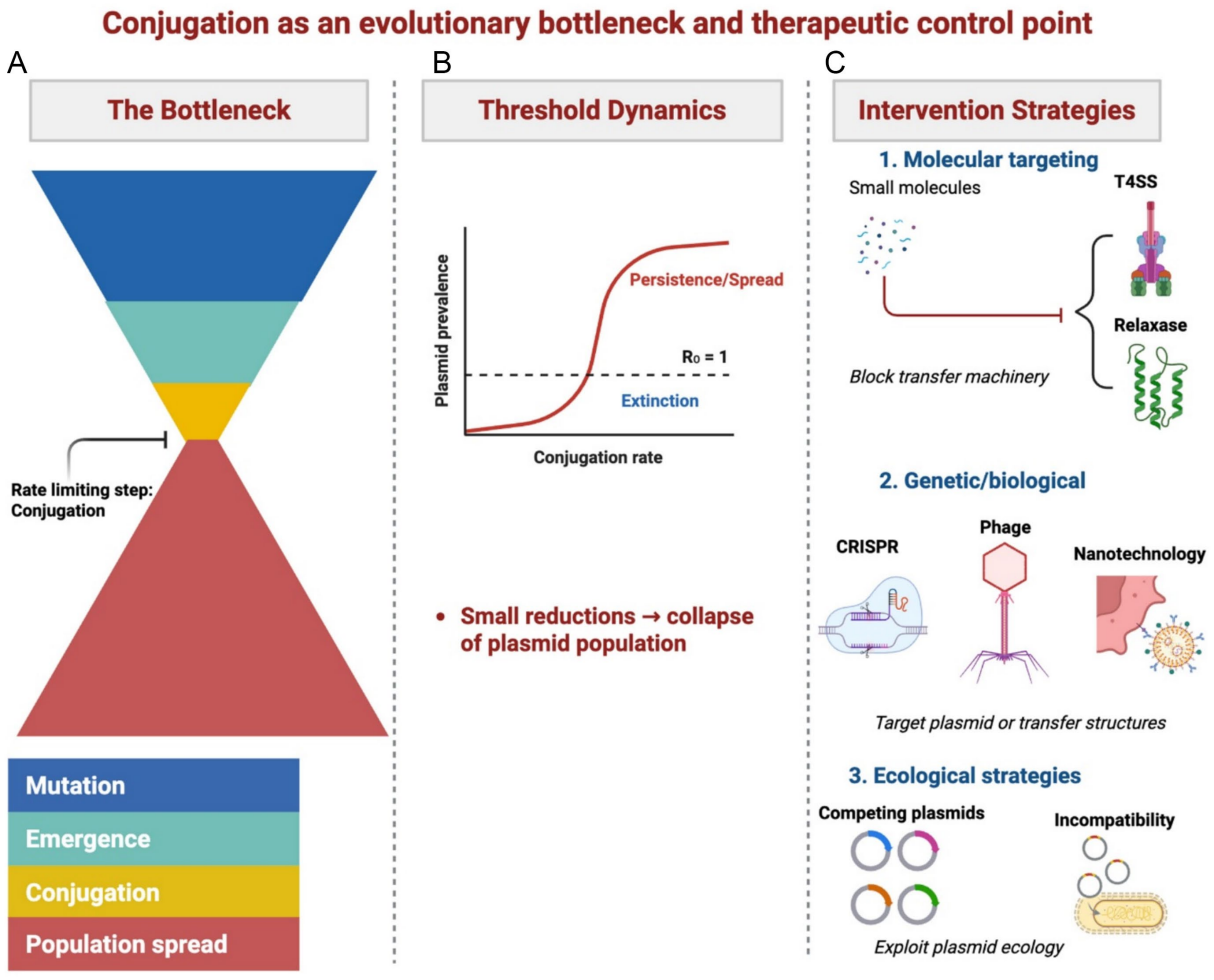
Conjugation as an evolutionary bottleneck and therapeutic control point. **(A)** Bottleneck architecture. Resistance emergence is decoupled from dissemination, which is constrained by conjugative transfer as a rate-limiting step governing amplification. **(B)** Threshold dynamics. Plasmid persistence depends on the plasmid reproductive number (R₀), with conjugation rate acting as a dominant control parameter. Small reductions in transfer efficiency can shift systems below the persistence threshold, leading to plasmid extinction. **(C)** Intervention strategies. Conjugation can be targeted at multiple levels, including inhibition of transfer machinery, sequence-specific plasmid targeting, and ecological disruption of plasmid maintenance. These interventions converge on reducing effective transfer rates. Evolutionary constraints limit escape, as resistance to inhibition often incurs trade-offs in transfer efficiency or host fitness. Together, these features position conjugation as a tractable control point for limiting antimicrobial resistance amplification.

In clinical contexts, these approaches could complement conventional antibiotics by limiting within-host plasmid dissemination, reducing cross-species transfer in microbiomes, and lowering the probability of emergence of multidrug-resistant infections. Collectively, these considerations position conjugation-targeted interventions not as standalone solutions but as components of integrated, evolution-aware therapeutic frameworks designed to limit resistance amplification rather than merely eliminate resistant cells (Figure 5).

## 8 Challenges and future directions

### 8.1 Quantitative gaps

Progress in understanding conjugation is limited by the absence of standardized, quantitative metrics. Transfer rates are typically measured under heterogeneous laboratory conditions, hindering comparability and obscuring general principles. High-throughput approaches, including barcoded plasmid libraries and community-scale tracking systems, will be essential to quantify conjugation in complex environments and link intracellular processes to population-level dissemination.

### 8.2 Evolutionary unknowns

The evolutionary response of plasmids to targeted interventions remains poorly resolved. Key questions include the rate and fitness cost of resistance to conjugation inhibitors and whether compensatory evolution can restore function without restoring sensitivity. Plasmid-wide CRISPR interference (CRISPRi) screens provide a scalable framework to map genetic determinants of transfer, fitness, and adaptation under selective pressure (Calvo-Villamañán et al., 2025).

### 8.3 Translational priorities

Advancing conjugation-targeted strategies requires a shift toward predictive, evolution-aware frameworks that integrate context-aware antibiotic use, disruption of within-host plasmid transmission, and adjunctive interventions that suppress transfer, destabilize plasmids, or increase their fitness cost (Koh et al., 2025; San Millan and MacLean, 2017). Priorities include structural resolution of the T4SS to guide inhibitor design, scalable discovery pipelines, improved delivery systems for CRISPR-based tools, and preclinical models that capture both infection and transmission dynamics. Parallel investment in global plasmid surveillance will be critical to guide targeting and monitor evolutionary escape.

Collectively, these advances enable a transition from reactive antimicrobial strategies to proactive control of resistance amplification.

## 9 Conclusions and outlook

Conjugation is a dominant amplifier of plasmid-mediated antimicrobial resistance, enabling the rapid global dissemination of resistance genes across the One Health continuum. Resistance to last-resort antibiotics, including carbapenems, colistin, and tigecycline, is increasingly plasmid-encoded, and the convergence of multiple determinants on single mobile elements has produced extensively drug-resistant pathogens with limited therapeutic options. Collectively, these observations support reframing antimicrobial resistance as an amplification problem, requiring interventions that target gene flow rather than gene emergence alone.

This duality positions conjugation as both driver and opportunity. While it underpins resistance spread, it also represents an evolutionary bottleneck a discrete and targetable step in the dissemination process. The molecular machinery of conjugation, its economic constraints, regulatory control, and ecological dependence together define a landscape of exploitable vulnerabilities.

Emerging strategies, including small-molecule inhibitors, CRISPR-based systems, nanobodies, pilus-targeting phages, and plasmid displacement approaches, demonstrate that conjugation can be suppressed. However, translation will require overcoming key challenges, including delivery, evolutionary escape, and integration into clinical and environmental frameworks. Effective implementation will depend on embedding these approaches within broader One Health strategies encompassing stewardship, infection control, and environmental management.

By reframing conjugation as a controllable parameter of resistance amplification, future interventions may shift the trajectory of antimicrobial resistance from expansion toward containment. The central challenge is no longer establishing the role of conjugation in resistance dissemination, but translating this mechanistic understanding into durable, evolution-aware therapeutic strategies.
